# Engineering a thermostable recombinant *Bacillus thuringiensis* serovar *tenebrionis* expressing a date palm dehydrin gene for improved red palm weevil biocontrol

**DOI:** 10.3389/fpls.2026.1938538

**Published:** 2026-10-07

**Authors:** Mughair Abdul Aziz, Shafeeq Rahman, Uday Kishore, Khaled Masmoudi

**Affiliations:** 1 Department of Integrative Agriculture, College of Agriculture and Veterinary Medicine, United Arab Emirates University, Al Ain, United Arab Emirates; 2 Department of Veterinary Medicine, College of Agriculture and Veterinary Medicine, United Arab Emirates University, Al Ain, United Arab Emirates

**Keywords:** biopesticide, date palm, heat stress, *PdDHN1*, red palm weevil

## Abstract

The efficacy of *Bacillus thuringiensis* based biopesticides under field conditions is frequently compromised due to the thermal degradation of δ-endotoxins at elevated temperatures. Dehydrins (DHNs) are highly hydrophilic and intrinsically disordered proteins characterized by flexible structures and the ability to interact with a wide range of cellular molecules, including proteins, enzymes, membranes, and ions. These properties enable DHNs to protect and stabilize cellular macromolecules and help maintain cellular functions under adverse environmental conditions. In this study, *B. thuringiensis* serovar *tenebrionis* was engineered to express the date palm *DHN* gene *PdDHN1*, and its performance was compared with wild-type and empty vector strains after defined thermal preconditioning at 28 °C and 45 °C for 7 days before application. After heat-stress preconditioning, the *PdDHN1*-expressing strain retained the highest sporulation capacity (16.5 × 10^7^ spores/ml), exceeding both the wild-type strain (11.8 × 10^7^ spores/ml) and the empty vector strain (9.2 × 10^7^ spores/ml). Bioassays against red palm weevil (RPW), *Rhynchophorus ferrugineus* showed that the recombinant strain had stronger insecticidal activity than the control strains under both thermal regimes, causing faster survival decline and greater mortality. *PdDHN1*-transformed formulations reduced RPW larval and adult survival to below 10% after 21 days, whereas the wild-type and empty-vector strains showed a lower reduction in RPW survival. Feeding efficiency assays showed physiological disruption in insects exposed to the *PdDHN1*-transformed *B. thuringiensis* strain; in larvae, feed conversion efficiency (FCE), efficiency of conversion of ingested food (ECI), and efficiency of conversion of digested food (ECD) decreased by 73.72%, 70.65%, and 66.86%, respectively, compared with wild-type and empty vector strains. In adults, ECI and ECD were reduced by 70.65% and 63.40%, respectively, under the same conditions. The expression of *PdDHN1* enhances *B. thuringiensis* thermal stability and insecticidal efficacy under heat stress, providing an approach to improve biopesticide performance in high-temperature environments.

## 1 Introduction

*Bacillus thuringiensis* is a Gram-positive spore-forming bacterium widely used as a biological insecticide because of its strong specificity toward target pests and environmentally sustainable mode of action. Its efficacy is primarily associated with a diverse array of toxins and virulence factors, including δ-endotoxins (Cry proteins), Cyt proteins, and vegetative insecticidal proteins (Vip), which collectively contribute to toxicity against a broad range of economically important insect pests (Haque et al., 2026). However, environmental stresses, especially high temperatures, can reduce the biological performance of *B. thuringiensis*. Heat exposure may affect the stability and activity of insecticidal components, potentially resulting in reduced insecticidal efficacy, for instance, storage at 35 ± 2 °C for extended periods has been shown to reduce *B. thuringiensis* toxicity by up to 60% in susceptible larvae (Moustafa et al., 2018). Because *B. thuringiensis*-based biocontrol agents may encounter elevated temperatures during storage, transportation, and field application, maintaining their biological activity under high-temperature conditions is important for their reliable use in warm environments. This indicates the need to investigate biological strategies that can enhance thermal tolerance and maintain the biological performance of *B. thuringiensis* under elevated temperature conditions (Wu et al., 2011).

On the other hand, plants have evolved sophisticated adaptive mechanisms that enable survival under environmental stresses that disrupt cellular homeostasis and impair growth. Abiotic stress conditions commonly trigger osmotic imbalance and the excessive accumulation of reactive oxygen species, leading to oxidative damage of proteins, membranes, and nucleic acids. To counteract these effects, plants activate stress-responsive pathways that drive the accumulation of protective proteins. Among these, dehydrins (DHNs), also known as group II late embryogenesis abundant (LEA) proteins, constitute a major class of stress-associated molecules. First identified in cotton seeds (Galau and Close, 1992), DHNs are now recognized to accumulate in vegetative tissues in response to a wide range of abiotic stresses, including dehydration, salinity, freezing, and heat stress (Amara et al., 2014; Hernandez-Sanchez et al., 2022). Structurally, DHNs are highly hydrophilic, intrinsically disordered proteins enriched in glycine and lysine residues and typically lacking cysteine and tryptophan. This flexible conformation enables interactions with diverse cellular components, including enzymes, membranes, ions, and nucleic acids, thereby stabilizing macromolecules and maintaining cellular integrity under stress conditions (Smith and Graether, 2022).

Structurally, DHNs are defined by conserved sequence motifs, including the K-, S-, and Y-segments, which determine their functional versatility under abiotic stress conditions. The K-segment is the most conserved and forms amphipathic α-helices that facilitate interactions with membranes and partially unfolded proteins, thereby directly contributing to macromolecular stabilization. The serine-rich S-segment is often phosphorylated and is implicated in subcellular targeting, while the Y-segment, typically located near the N-terminus, is associated with stress-responsive regulation (Richard Strimbeck, 2017; Smith and Graether, 2022). These structural features enable DHNs to function as molecular stabilizers by maintaining hydration shells, preventing protein aggregation, and preserving enzymatic activity under adverse environmental conditions. Consistent with this protective role, DHN expression is strongly induced by abiotic stress, and heterologous overexpression has been shown to enhance stress tolerance across diverse biological systems. For example, overexpression of wheat *DHN5*, rice *Rab16A*, and maize *Rab17* improves tolerance to drought, salinity, and osmotic stress (Cheng et al., 2002; Figueras et al., 2004; Brini et al., 2007). DHNs have also been shown to directly protect enzyme function under thermal and oxidative stress by stabilizing proteins such as lactate dehydrogenase, catalase, and glutathione transferases (Liu et al., 2017; Nguyen et al., 2020; Ohkubo et al., 2020). Our previous studies demonstrated that DHNs can directly protect enzyme function under stress conditions by stabilizing proteins such as lactate dehydrogenase and β-glucosidase (Abdul Aziz et al., 2023; Abdul Aziz et al., 2025; Brini et al., 2010).

The incorporation of plant-derived stress-responsive proteins into microbial systems provides a potential strategy for enhancing their tolerance to environmental stress and maintaining biological performance under adverse conditions. In the present study, the date palm *DHN* gene *PdDHN1* was introduced into *B. thuringiensis* serovar *tenebrionis* to investigate its contribution to bacterial performance following prolonged thermal exposure. Given the reported protein- and membrane-protective properties of DHNs, *PdDHN1* expression may help bacterial cells maintain physiological functions under elevated temperature conditions. Accordingly, evaluation of bacterial growth and sporulation following thermal exposure, together with subsequent assessment of insecticidal activity, provides an integrated approach for examining the effects of *PdDHN1* expression on the thermal tolerance and biological performance of *B. thuringiensis*. This strategy may contribute to the development of *B. thuringiensis* strains with improved performance under high-temperature conditions.

The improved biological performance of *B. thuringiensis* strains may provide a useful approach for managing *Rhynchophorus ferrugineus*, commonly known as the red palm weevil (RPW), which poses a severe threat to *Phoenix dactylifera* (date palm), a crop largely cultivated under high-temperature arid conditions. Adult females of RPW oviposit in wounds, fissures, and cracks of palm tissue (Manee et al., 2023), while the larval stage is the most destructive, tunneling deeply into the trunk and forming extensive galleries that lead to frond deformation and eventual palm mortality (Mogahed, 2023). The warm climate of date palm-growing regions further accelerates pest development, intensifying infestation pressure and complicating management strategies. Although RPW is an invasive pest, effective natural enemies are limited, and reliance on chemical control remains environmentally and economically unsustainable. Naturally occurring microbial and biological control agents have also shown limited and inconsistent success in RPW management in date palm plantations (Kassem et al., 2020), partly because their persistence and efficacy can be adversely affected by high temperatures, ultraviolet radiation, and other harsh environmental conditions. Moreover, the cryptic feeding behavior of RPW larvae deep within palm tissues can limit the effective delivery and activity of microbial agents at the target site. These limitations indicate the need for biologically enhanced strategies capable of maintaining *B. thuringiensis* efficacy under thermally stressful field conditions.

The present study aimed to genetically transform *B. thuringiensis* serovar *tenebrionis* to heterologously express the date palm *DHN* gene, *PdDHN1*, and to investigate its potential to enhance the thermal tolerance and biological performance of *B. thuringiensis* under heat stress conditions. The effects of *PdDHN1* expression were evaluated through bacterial growth and sporulation under control and elevated temperature conditions. Furthermore, the insecticidal performance of the transformed and non-transformed strains was assessed against RPW, focusing on dose-dependent mortality, time-dependent survival patterns, and efficacy under thermal stress. Additionally, nutritional indices of the target insect, including feeding conversion efficiency, were evaluated to determine the physiological impact of the recombinant strain on insect development and survival.

## 2 Materials and methods

### 2.1 Construction of *PdDHN1* recombinant shuttle vector

To enable heterologous expression of the target gene in *B. thuringiensis* serovar *tenebrionis* (BGSC No. 4AA1), the coding sequence of the date palm DHN gene *PdDHN1* (XM_008800003.2) was amplified using gene-specific primers with *BamH*I and *EcoR*I restriction sites added (underlined sequences) at the 5′ and 3′ ends, respectively (5’-GGATCCATGGCGGAGGAGCAGAAG-3’ and 5’-GAATTCCTAGTGGGTAGCACCGGC-3’). The amplified PCR product was digested with the corresponding restriction enzymes and ligated into the Bacillus–*Escherichia coli* shuttle vector pHPS9 (ECE51:pHPS9), which had been digested with the same enzymes. The pHPS9 vector contains the P59 promoter, which drives transcription of the inserted gene within the expression cassette. The recombinant constructs were transformed into *E. coli* DH5α, screened, and confirmed by restriction digestion analysis to verify successful insertion and correct orientation of *PdDHN1* within the vector backbone. Plasmid DNA was purified using the QIAprep Spin Miniprep Kit (QIAGEN, Germany), following the manufacturer’s instructions, to obtain high-quality recombinant plasmid for downstream applications.

### 2.2 Bacterial strains, growth media, andculture conditions

*B. thuringiensis* serovar *tenebrionis* was used as the host strain for transformation and bioassays, while *E. coli* DH5α served as the cloning host for plasmid propagation. The shuttle vector pHPS9 was used for heterologous expression of *PdDHN1* in *B. thuringiensis*. Bacterial cultures were maintained on LB agar and grown in LB broth at 30 °C with shaking at 200 rpm. For culture preparation, a single colony was inoculated into LB broth and incubated overnight at 30 °C with shaking (Zhu et al., 2022). The resulting culture was used to inoculate fresh LB broth for subsequent experiments. Recombinant *B. thuringiensis* strains were cultured under the same conditions with the appropriate antibiotics for plasmid maintenance. Chloramphenicol and erythromycin were used for selection and maintenance of the recombinant plasmid during cloning and transformation procedures.

### 2.3 Transformation of *B. thuringiensis* serovar *tenebrionis*

Transformation of *B. thuringiensis* serovar *tenebrionis* was performed by electroporation following Macaluso and Mettus (1991). An overnight culture grown in LB medium at 30 °C was chilled on ice and harvested by centrifugation. Cells were washed twice with sterile ice-cold distilled water to remove residual salts and resuspended in electroporation buffer (0.625 M sucrose, 1 mM MgCl_2_). Aliquots of 100 µl competent cells were mixed with 0.5–1.0 µg of either recombinant plasmid DNA (pHPS9-PdDHN1) or empty plasmid (pHPS9) and transferred into a pre-chilled 0.2 cm electroporation cuvette (Invitrogen). The cell–DNA mixture was incubated on ice for 5–10 min before electroporation.

Electroporation was performed at 2.0 kV to 2.5 kV, corresponding to a field strength of 10 kV/cm to 12.5 kV/cm, using 25 µF capacitance and 200 Ω resistance, resulting in a time constant of approximately 4 ms to 6 ms. Immediately after pulsing, 2 ml of pre-warmed LB medium was added, and cells were allowed to recover at 30 °C for 1 h with gentle shaking. Transformants were selected on LB agar supplemented with chloramphenicol (25 µg ml^−1^) and erythromycin (15 µg ml^−1^) and incubated until colony formation. Putative transformants were confirmed through antibiotic selection and PCR amplification targeting the *PdDHN1* gene.

### 2.4 PCR amplification of *CryIIIA* gene

*B. thuringiensis* serovar *tenebrionis* strains (wild-type, empty vector, and *PdDHN1*-transformed) were screened for the presence of the insecticidal *cryIIIA* gene. Genomic DNA was extracted from bacterial cultures using the QIAGEN DNA (QIAGEN, Hilden, Germany) extraction protocol according to the manufacturer’s instructions (van Tongeren et al., 2011). The purified DNA served as template for PCR amplification using gene-specific primers col1A (5′-GTCCGCTGTATATTCAGGTG-3′) and col1B (5′-CACTTAATCCTGTGACGCCT-3′), which amplify a 649 bp fragment of the *cryIIIA* gene of *B. thuringiensis* serovar tenebrionis (Carozzi et al., 1991). PCR amplification was performed using Phusion Master Mix under standard cycling conditions with initial denaturation at 95 °C for 2 min, followed by 35 cycles of denaturation at 95 °C for 30 s, annealing at 57 °C for 30 s, and extension at 72 °C for 1 min, with a final extension step at 72 °C for 5 min. Amplified products were observed on a 1% agarose gel and visualized to confirm the expected amplicon size.

### 2.5 Expression analysis of *PdDHN1* by RT-PCR

For transcript analysis, total RNA was extracted from bacterial cultures (wild-type, empty vector, and *PdDHN1*-transformed) using the QIAGEN RNeasy kit according to the manufacturer’s instructions. cDNAs were prepared using M-MLV reverse transcriptase (Invitrogen) in accordance with the manufacturer’s recommendations and using a reverse primer without restriction site added (5’-CTAGTGGGTAGCACCGGC-3’). The synthesized cDNAs were used as template to amplify the transcripts of *PdDHN1* with the following primers (5’-ATGGCGGAGGAGCAGAAG-3’ and 5’-CTAGTGGGTAGCACCGGC-3’). PCR reactions were performed using Phusion Master Mix under standard cycling conditions with initial denaturation at 95 °C for 2 min, followed by 30 cycles of denaturation at 95 °C for 30 s, annealing at 55 °C for 30 s, and extension at 72 °C for 1 min, with a final extension step at 72 °C for 5 min. Amplified products were observed on a 1% agarose gel and visualized to confirm the expected amplicon size.

### 2.6 Total soluble protein quantification of *B. thuringiensis* serovar *tenebrionis* strains

Total soluble protein was quantified from the spore-crystal preparations of wild-type, empty-vector, and *PdDHN1*-expressing *B. thuringiensis* strains. Because the spore-crystal preparations contain crystalline protein inclusions, the samples were washed and subjected to alkaline solubilization before protein analysis. For each sample, 1 mL of the bacterial preparation was centrifuged at 10,000 × *g* for 10 min to collect the spore-crystal complex. The supernatant was discarded, and the pellet was washed twice with 1 mL of ice-cold 1 M NaCl to remove extracellular proteases and residual medium components, followed by two washes with distilled water. The final pellet was resuspended in alkaline carbonate buffer (50 mM Na_2_CO_3_, pH 10.5) containing 10 mM DTT as a reducing agent. The suspension was incubated at 37 °C for 2 h with constant shaking to facilitate solubilization of the protein-containing crystalline fraction. Following incubation, the samples were centrifuged at 12,000 × *g* for 15 min and the resulting supernatant containing the solubilized proteins was collected for protein analysis.

Total soluble protein concentration in the alkaline-extracted preparation was determined using the Bradford assay with Coomassie Brilliant Blue G-250 dye by measuring absorbance at 595 nm (Bradford, 1976). The protein concentration was expressed as µg mL^−1^ and used to compare the total soluble protein content of the preparations obtained from wild-type, empty-vector, and *PdDHN1*-expressing *B. thuringiensis* strains following the different thermal exposure conditions. SDS-PAGE was used separately to examine the protein profiles of the alkaline-extracted preparations.

### 2.7 Thermal preconditioning of *B. thuringiensis* serovar *tenebrionis* strains

To evaluate the effect of prolonged thermal exposure on bacterial performance, *B. thuringiensis* strains (wild-type, empty vector, and *PdDHN1*-transformed) were subjected to controlled thermal preconditioning prior to sporulation analysis and insect bioassays. Vegetative bacterial cells from actively growing cultures were used as the initial inoculum in 2×SG medium. All bacterial cultures were initially grown at 28 °C. Following growth, the cultures were stored to thermal preconditioning at 28 °C (control condition) or 45 °C (heat-stress condition) for 7 days. No antibiotics were added during the 7-day thermal preconditioning period. Cultures were maintained under continuous shaking to ensure aeration and uniform exposure to the respective temperature conditions. The thermal preconditioning was designed as a controlled experimental heat-stress treatment to compare the ability of the different *B. thuringiensis* strains to maintain the stability and activities of Cry proteins.

### 2.8 Estimation of spore production in *B. thuringiensis* serovar *tenebrionis* strains

Spore production in *B. thuringiensis* serovar *tenebrionis* strains was determined by quantifying heat-resistant viable spores. Briefly, 1 ml of fully sporulated culture was collected from 2×SG medium following completion of sporulation and autolysis. To selectively enumerate spores, samples were subjected to a standardized heat treatment at 80 °C for 10 min, which eliminates vegetative cells while preserving heat-resistant spores. Following heat treatment, serial dilutions were prepared in sterile physiological saline, and aliquots were plated on nutrient agar. For recombinant strains, media were supplemented with chloramphenicol (25 µg ml^−1^) and erythromycin (15 µg ml^−1^) to maintain plasmid selection pressure. Plates were incubated at 28 °C for 20 h to 24 h, and colony-forming units (CFUs) were counted to estimate viable spore concentration. Spore counts were expressed as spores per milliliter of culture (CFU/ml equivalent).

### 2.9 Light microscopy analysis of *B. thuringiensis* serovar *tenebrionis* strains

*PdDHN1*-transformed and non-transformed cultures of *B. thuringiensis* serovar *tenebrionis* were examined following thermal preconditioning at 28 °C and 45 °C to assess temperature-associated changes in cellular morphology. Bacterial cultures were prepared on clean glass slides, air-dried, and heat-fixed. The smears were stained with Coomassie Brilliant Blue R-250. Excess stain was gently removed, and slides were air-dried prior to microscopic examination. Samples were examined by bright-field light microscopy using a 100× oil-immersion objective with immersion oil using a VxL-Bino (Almicro) microscope. Cellular morphology, including cell shape and observable heat-associated morphological changes, was evaluated for the transformed and non-transformed strains to assess the effects of temperature on cellular morphology.

### 2.10 Collection and rearing of RPW larva and adult insects

Larvae of the RPW were collected from infested, untreated date palm trees in Al Ain, United Arab Emirates, following standard phytosanitary cutting procedures for pest containment and field management. Actively feeding third instar larvae within a comparable body weight range (2.5 g to 3.5 g g) were selected (**Figures 1A, B**). Adult insects were collected using pheromone-baited traps deployed in infested date palm plantations (**Figure 1C**). All specimens were transported to the laboratory in aerated plastic containers supplied with fresh palm tissue to maintain viability during transport. In the laboratory, larvae and adults were maintained under controlled environmental conditions and reared on fresh sugarcane pieces until use in experiments.

**Figure 1:**
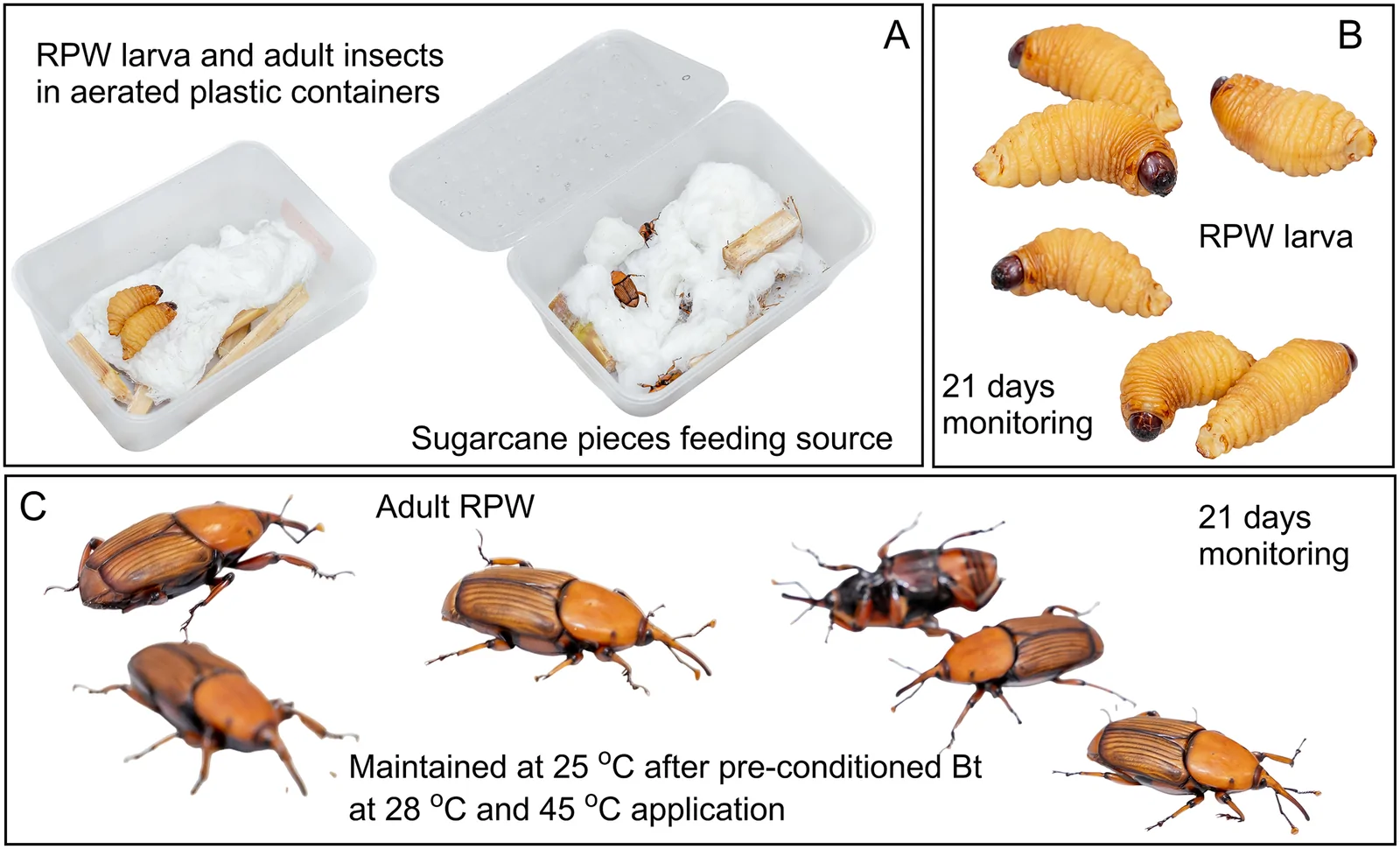
RPW larva and adult insects for mortality bioassay. **(A)** RPW larva and adult insects transported through aerated plastic containers. RPW **(B)** larva and **(C)** adult insects monitored for 21 days after *B. thuringiensis* treatments.

### 2.11 RPW bioassay of *B. thuringiensis* formulated biopesticide

Formulated suspensions of wild-type *B. thuringiensis*, empty vector control, and *PdDHN1*-transformed strains were prepared for bioassay evaluation. Prior to application, bacterial cultures were subjected to thermal preconditioning at either 28 °C (control condition) or 45 °C (heat-stress condition) for one week to assess the effect of prior thermal exposure on insecticidal performance. Bioassays were conducted at room temperature (25 °C) and 65% relative humidity using standardized spore-containing bacterial suspensions. A dose–response range of 10^3^ to10^8^ spores (CFU mL^−1^ equivalent) was prepared for each treatment based on viable spore counts to determine lethal concentration values (LC_50_ and LC_90_) through probit analysis using RStudio version 4.6.1.

A discriminating concentration of 6.5 × 10^6^ CFU mL^−1^ was selected for the survival and physiological bioassays based on the probit-derived LC_50_ and LC_90_ estimates and preliminary dose–response assays. In the preliminary dose–response assays, bacterial suspensions ranging from 10^3^ to 10^8^ CFU mL^−1^ were prepared from viable cultures and used to establish concentration-dependent mortality responses for the different *B. thuringiensis* strains and insect stages. Based on these results, 6.5 × 10^6^ CFU mL^−1^ was selected as a common discriminating concentration for subsequent comparative bioassays.

For treatment preparation, bacterial cultures were adjusted to the required concentration using viable spore counts, and the respective suspensions were maintained under uniform conditions before application. Fresh sugarcane pieces were used as the feeding substrate for both larval and adult RPW. Sugarcane pieces of comparable size were completely immersed in the corresponding bacterial suspension for 30 min to allow treatment uptake. Following dipping, the pieces were removed aseptically and air-dried until the excess suspension had evaporated before being introduced into the insect containers. Separate sugarcane pieces were prepared for the untreated control, buffer/formulation-only control, empty-vector *B. thuringiensis*, wild-type *B. thuringiensis*, and *PdDHN1*-transformed *B. thuringiensis* treatments.

For the larval bioassay, individually selected third-instar RPW larvae were provided with sugarcane pieces treated with the respective bacterial suspensions. Adult RPW were similarly exposed to treated sugarcane pieces under the same bacterial concentration and treatment conditions. Larvae and adults were maintained separately under controlled laboratory conditions, and fresh treated feeding material was provided as required throughout the observation period. Each treatment consisted of three independent biological replicates, with 30 insects per replicate.

Mortality was assessed daily for 21 days after treatment. An insect was considered dead when it showed no movement or response to gentle physical stimulation. The 21-day observation period was used to capture both early and delayed mortality responses following ingestion of the treated feeding substrate and to characterize the cumulative insecticidal effects of the different *B. thuringiensis* strains. Survival data were recorded throughout the observation period and analyzed using Kaplan–Meier survival analysis.

### 2.12 Determination of feeding efficiency and nutrient conversion in larval and adult stages

For each treatment, individuals were provided with a pre-weighed quantity of food, and their initial body weight was recorded using a precision analytical balance (QEC-Series, Qualitest, USA). After the experimental period, the remaining uneaten food was collected and weighed to determine food consumption. The final body weight of each individual was measured to calculate weight gain. Fecal matter produced during the experiment was collected and air-dried to constant weight before weighing to accurately estimate the amount of undigested food. Food ingested was calculated as the difference between the food supplied and the leftover food, while digested food was obtained by subtracting the dry weight of feces from the total food ingested. Weight gain was determined as the difference between final and initial body weight. The nutritional indices were calculated using the following standard formulas:


FCE(%)=(Weight gain÷Food consumed)×100

ECI(%)=(Weight gain÷Food ingested)×100

ECD(%)=(Weight gain÷Food digested)×100

### 2.13 Statistical analysis

Data obtained from mortality assays, larval weight gain, feed conversion ratio (FCR), and adult survival were analyzed using appropriate statistical methods. Differences among treatments involving wild-type, empty vector, and *PdDHN1*-recombinant *B. thuringiensis* serovar *tenebrionis* under different temperature conditions were assessed using one-way analysis of variance (ANOVA), followed by Tukey’s *post hoc* test for multiple comparisons. Prior to ANOVA, data were evaluated for the assumptions of normality and homogeneity of variance using Shapiro–Wilk and Levene’s tests, respectively. Results are presented as mean ± standard error (SE), and statistical significance was considered at p< 0.05. Dose–response mortality data were analyzed using probit regression to estimate lethal concentrations (LC_50_ and LC_90_). Probit models were fitted using a generalized linear model (GLM) with a binomial error distribution and probit link function in R software (version 4.6.1). Probit analyses were conducted separately for each strain (wild-type, empty vector, and *PdDHN1*-transformed *B. thuringiensis*), developmental stage (larva and adult), and temperature condition (28 °C and 45 °C). Model performance was evaluated using residual deviance and Akaike Information Criterion (AIC). Treatment differences were inferred based on non-overlapping confidence intervals of LC estimates. LC_50_ and LC_90_ values were estimated from fitted models using the MASS package, with standard errors calculated for each estimate. Survival data were analyzed using Kaplan–Meier survival analysis, and differences among treatment groups were evaluated using log-rank (Mantel–Cox) tests. Pearson correlation analysis was performed to evaluate relationships among physiological, biological parameters, and other measured traits. Correlation coefficients (r) and associated significance levels were calculated, with p< 0.05 considered statistically significant.

## 3 Results

### 3.1 Molecular cloning and confirmation of *PdDHN1*-expressing *B. thuringiensis* serovar *tenebrionis*

The recombinant plasmid pHPS9-*PdDHN1* was constructed and confirmed by restriction digestion, which produced DNA fragments with the expected sizes of the vector backbone and *PdDHN1* insert, verifying correct plasmid assembly (**Figures 2A, B**). Transformation of *B. thuringiensis* serovar *tenebrionis* with the recombinant construct was further validated by PCR. Amplification with *cryIIIA*-specific primers (col1A and col1B) produced the expected 649 bp band in the recombinant strain, confirming retention of the native insecticidal gene (**Figure 2C**). RT-PCR analysis further showed strong *PdDHN1* transcript accumulation in the pHPS9-*PdDHN1* recombinant strain, indicatin successful expression of the introduced construct (**Figure 2D**). The absence of expression of *PdDHN1* gene in the wild-type and empty vector strains, together with consistent detection of cryIIIA, supports the specificity and stability of the transformation.

**Figure 2:**
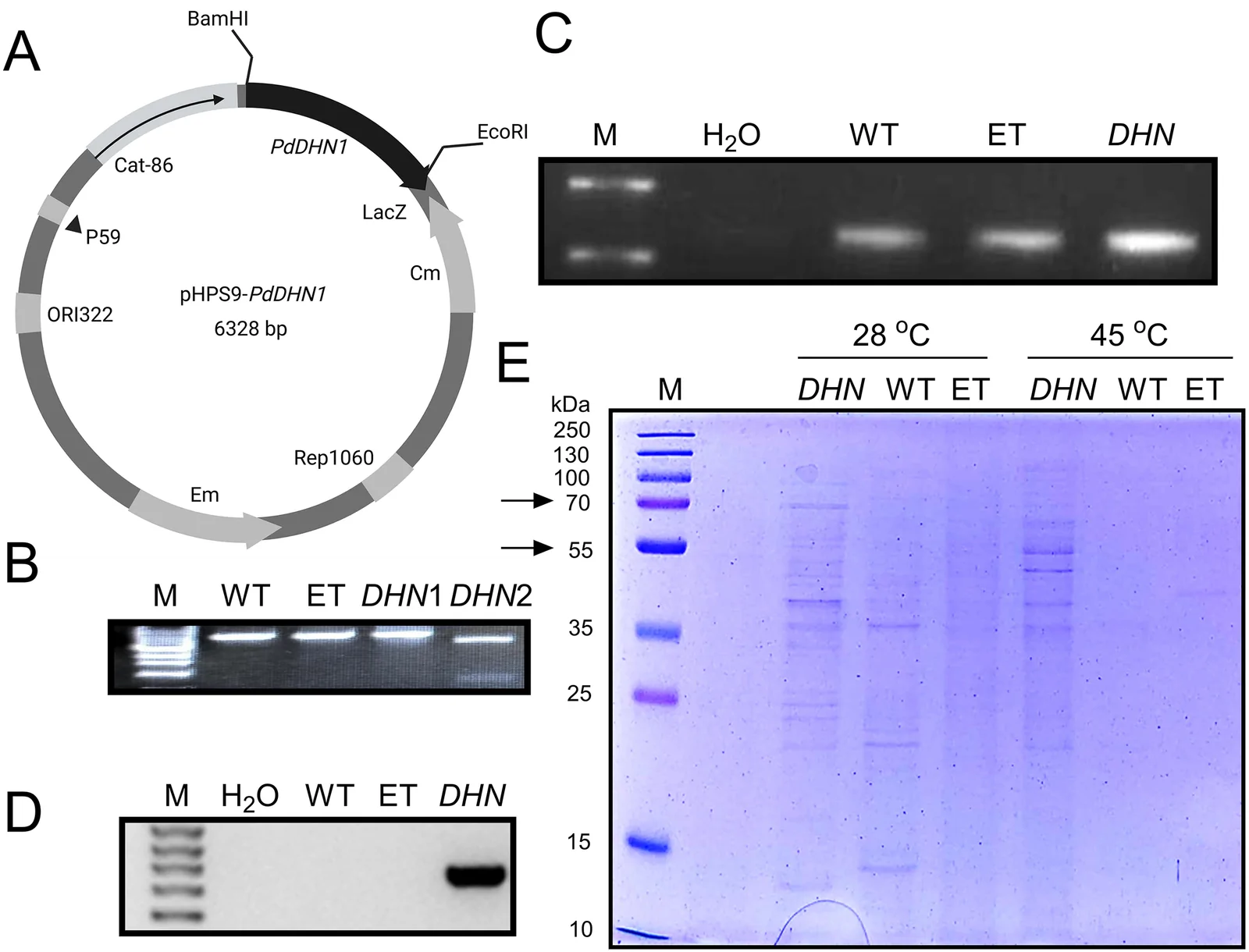
Construction of Bacillus-*E. coli* recombinant shuttle vector (pHPS9-*PdDHN1*). **(A)** Gene map of recombinant shuttle vector with *PdDHN1* gene. **(B)** Agarose gel with the restriction digestion of *PdDHN1* gene. **(C)** PCR amplification of *CryIIIA* gene in *B. thuringiensis* strains. **(D)** *PdDHN1* transcript analysis with RT-PCR in transformed and non-transformed *B. thuringiensis* strains. **(E)** SDS-PAGE analysis of *B. thuringiensis* extracts. M; ladder, WT- wild-type *B. thuringiensis*, ET - empty plasmid *B. thuringiensis*, and *DHN* – *PdDHN1*-transformed *B. thuringiensis*. Arrows indicate predicted size range of Cry3Aa between 66 kDa and 73 kDa.

SDS-PAGE was performed to compare the protein profiles of wild-type, empty-plasmid, and *PdDHN1*-transformed *B. thuringiensis* cultures maintained at 28 °C and 45 °C. The three bacterial extracts displayed differences in the banding patterns under the two temperature conditions (**Figure 2E**). At 28 °C, the wild-type and empty vector extracts showed broadly similar protein profiles, whereas the *PdDHN1*-transformed *B. thuringiensis* extract exhibited additional and more obvious protein bands across the resolved molecular-weight range. Following incubation at 45 °C, changes in band intensity were observed in all extracts, indicating temperature-associated alterations in the bacterial protein profile. However, the *PdDHN1*-transformed *B. thuringiensis* extract retained several prominent protein bands at 45° (**Figure 2E**).

### 3.2 Total soluble protein of *B. thuringiensis* strains following thermal exposure

Total soluble protein content was determined for wild-type, empty-vector, and *PdDHN1*-expressing *B. thuringiensis* preparations at three experimental stages including immediately after initial culture growth at 28 °C (post-growth, Day 0), following 1 week of maintenance at 28 °C, and following 1 week of exposure to 45 °C (**Figures 3A–C**). At the post-growth stage, the total soluble protein concentrations were 70.25 µg/ml, 68.43 µg/ml, and 89.86 µg/ml for wild-type, empty-vector, and *PdDHN1*-expressing *B. thuringiensis*, respectively (**Figure 3A**). Following 1 week at 28 °C, the corresponding concentrations were 68.43 µg/ml, 66.78 µg/ml, and 91.29 µg/ml for wild-type, empty-vector, and *PdDHN1*-expressing *B. thuringiensis*, respectively (**Figure 3B**). After 1 week of exposure to 45 °C, the total soluble protein concentrations were 39.14 µg/ml for wild-type, 38.25 µg/ml for empty vector, and 95.14 µg/ml for *PdDHN1*-expressing *B. thuringiensis* (**Figure 3C**).

**Figure 3:**
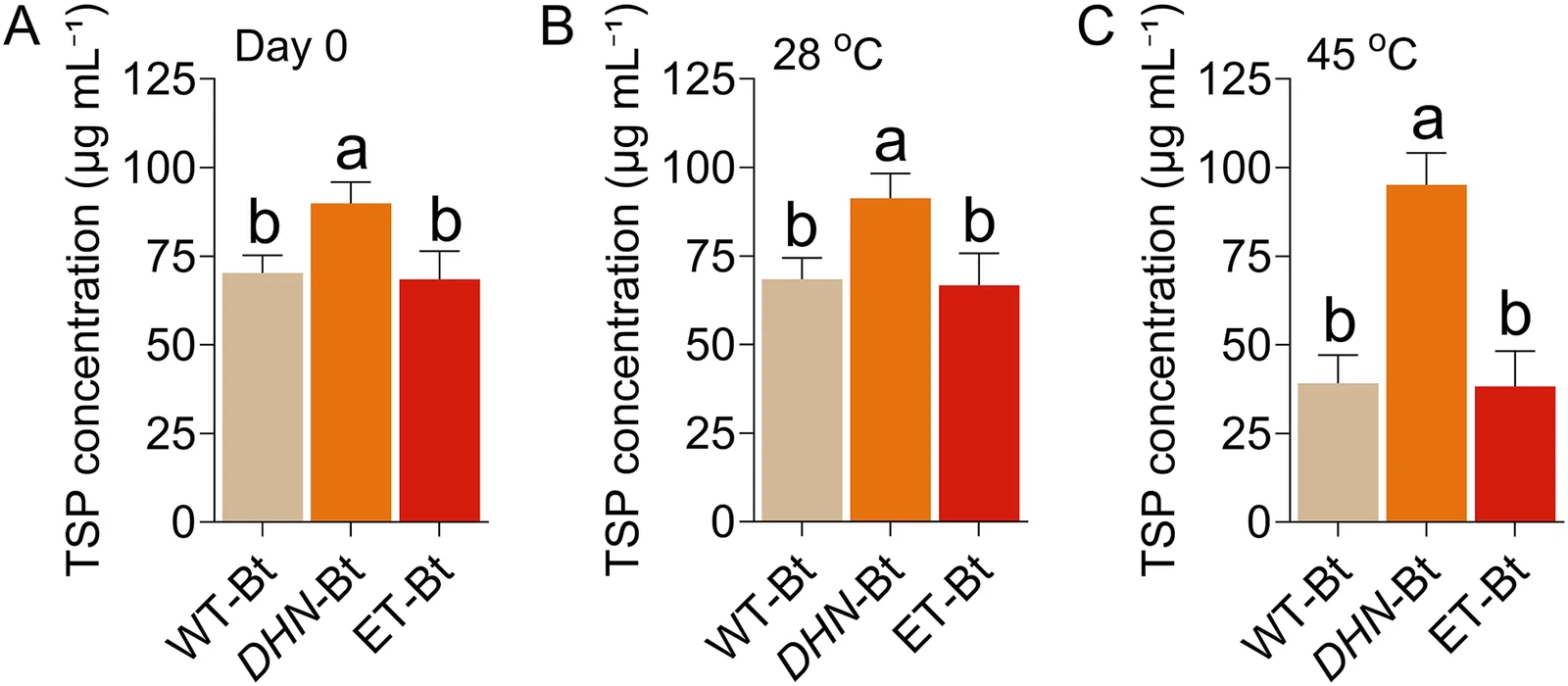
Total soluble protein concentration of *B. thuringiensis* serovar *tenebrionis* at different stages of thermal exposure. **(A)** Post-growth (Day 0). **(B)** 1 week at 28 °C. **(C)** 1 week at 45 °C. Values are presented as mean ± SE. Different letters indicate statistically significant differences among treatments (p< 0.05), based on one-way ANOVA followed by Tukey’s *post hoc* test. WT-Bt, wild-type *B. thuringiensis*; ET-Bt, empty-plasmid *B. thuringiensis*; *DHN*-Bt, *PdDHN1*-transformed *B. thuringiensis*.

The *PdDHN1*-transformed *B. thuringiensis* preparation showed the highest total soluble protein concentration at all three sampling stages, with the concentration increasing from 89.86 µg/ml at post-growth (Day 0) to 91.29 µg/ml following 1 week at 28 °C and 95.14 µg/ml following exposure to 45 °C. In contrast, wild-type *B. thuringiensis* showed a gradual decrease in total soluble protein concentration from 70.25 µg/ml at post-growth to 68.43 µg/ml after 1 week at 28 °C and 39.14 µg/ml following exposure to 45 °C. Empty vector-transformed *B. thuringiensis* similarly decreased from 68.43 µg/ml at post-growth to 66.78 µg/ml at 28 °C and 38.25 µg/ml at 45 °C. These results indicate that *PdDHN1* expressing *B. thuringiensis* maintained a higher total soluble protein concentration following prolonged exposure to 45 °C compared with wild-type and empty plasmid vector *B. thuringiensis*.

### 3.3 Microscopy of *B. thuringiensis* serovar *tenebrionis* strains

Light microscopy was used to examine the morphology of wild-type, empty-vector, and *PdDHN1*-expressing *B. thuringiensis* serovar *tenebrionis* cultures following the 7-day thermal exposure. At 28 °C, all three strains showed similar microscopic appearance, with bacterial cells distributed individually and in small clusters (**Figure 4**). Following exposure to 45 °C, differences in the overall distribution and appearance of the cells were observed, particularly in the wild-type *B. thuringiensis* and empty vector preparations, which showed a less distinct cellular appearance. The *PdDHN1*-expressing *B. thuringiensis* serovar *tenebrionis* preparation showed comparatively fewer major changes in cellular appearance under the same thermal conditions. These observations indicate differences in the microscopic appearance of the bacterial cultures following prolonged thermal exposure.

**Figure 4:**
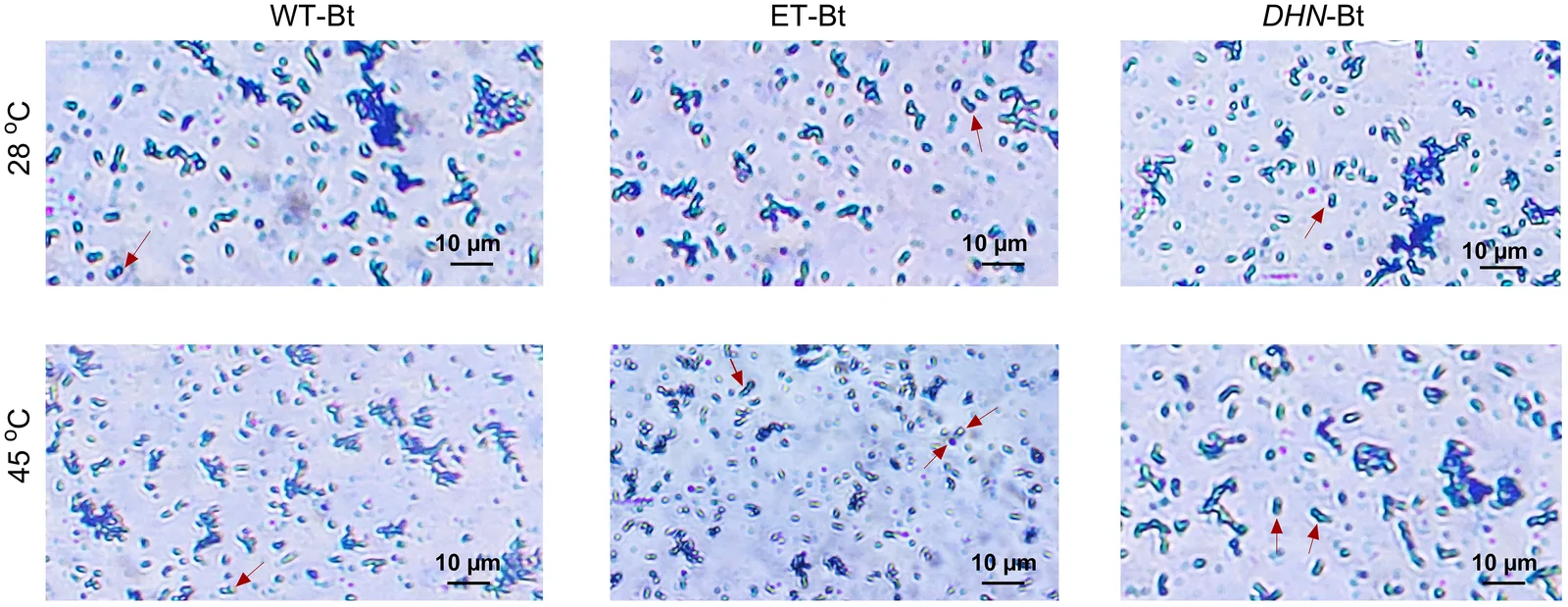
Light microscopy of *B. thuringiensis* strains at normal and heat stress conditions. Microscopic analysis of wild-type (WT), empty vector (ET), and *PdDHN1*-transformed (*DHN*-Bt) *B. thuringiensis* serovar *tenebrionis* observed at 100× oil-immersion magnification. Arrows indicate spores and/or crystals.

### 3.4 Spore production in different *B. thuringiensis* serovar *tenebrionis* strains

Spore production was quantified following the 7-day thermal exposure to evaluate the effects of temperature on the sporulation response of wild-type, empty vector, and *PdDHN1*-expressing *B. thuringiensis* strains. Under the 28 °C control condition, all strains exhibited substantial spore production, with relatively small differences among treatments. The *PdDHN1*-transformed strain produced 18.6 × 10_7_ spores/ml, followed by the wild-type strain at 17.8 × 10^7^ spores/ml, while the empty-vector strain produced 16.9 × 10^7^ spores/ml (**Table 1**).

**Table 1:** Spore yields for wild-type and recombinant *B. thuringiensis* serovar *tenebrionis* strains.

Strain	28 ^o^C	45 ^o^C
Wild type Bt	17.8 ± 1.62^a^ × 10^7^ spores/ml	11.8 ± 1.53^b^ × 10^7^ spores/ml
Empty plasmid Bt	16.9 ± 1.85^b^ × 10^7^ spores/ml	9.2 ± 1.17^c^ × 10^7^ spores/ml
*DHN*-transformed Bt	18.6 ± 2.60^a^ × 10^7^ spores/ml	16.5 ± 1.85^a^ × 10^7^ spores/ml

Exposure to 45 °C resulted in significant differences in spore production among the bacterial strains (p< 0.001). Spore production decreased to 11.8 × 10^7^ spores/ml in the wild-type strain and 9.2 × 10^7^ spores/ml in the empty-vector strain. In contrast, the *PdDHN1*-transformed strain maintained a high spore yield of 16.5 × 10^7^ spores/ml, with no significant reduction relative to its corresponding 28 °C treatment. Under the 45 °C condition, the *PdDHN1*-transformed strain produced significantly more spores than both the wild-type and empty-vector strains (**Table 1**). This shows that the three bacterial strains differed in their sporulation response following prolonged exposure to 45 °C, with the *PdDHN1*-expressing strain maintaining a higher spore yield under the imposed thermal-stress condition. This result indicates an association between *PdDHN1* expression and maintenance of sporulation capacity under the experimental heat-stress conditions.

### 3.5 Lethal concentration responses of *B. thuringiensis* serovar *tenebrionis* strains across RPW stages and temperature conditions

Larval bioassays conducted at 25 °C following application of *B. thuringiensis* formulations preconditioned at either 28 °C or 45 °C revealed strong dose–response relationships across all strains and treatments, with all probit regression models being highly significant (p< 0.001). These results confirm consistent insecticidal activity under standardized post-application conditions, while highlighting the effect of bacterial thermal preconditioning on toxicity. At 28 °C preconditioning, clear strain-dependent differences in larvicidal potency were observed (**Table 2**). The *PdDHN1*-transformed strain exhibited the highest toxicity, with the lowest LC_50_ (3.99 ± 0.19) and LC_90_ (5.82 ± 0.27), followed by the wild-type strain (LC_50_ - 5.07 ± 0.22 and LC_90_ - 7.67 ± 0.39), while the empty vector strain showed reduced efficacy (LC_50_ - 5.80 ± 0.26 and LC_90_ - 9.01 ± 0.60).

**Table 2:** Toxicity of *B. thuringiensis* strains against RPW larva under normal and heat stress conditions.

Strain	Temp ^o^C	Stage	LC_50_	LC_90_	LC_50_ CFU	LC_90_ CFU
WT	28	Larva	5.07 ± 0.22	7.67 ± 0.39	1.17×10^5^	4.68×10^7^
WT	45	Larva	6.06 ± 0.26	9.20 ± 0.61	1.15×10^6^	1.60×10^9^
ET	28	Larva	5.80 ± 0.26	9.01 ± 0.60	6.36×10^5^	1.02×10^9^
ET	45	Larva	7.11 ± 0.37	10.75 ± 0.95	1.27×10^7^	5.56×10^10^
DHN	28	Larva	4.26 ± 0.20	6.30 ± 0.29	1.82×10^4^	1.99×10^6^
DHN	45	Larva	3.99 ± 0.19	5.82 ± 0.27	9.78×10^3^	6.65×10^5^

When *B. thuringiensis* formulations were preconditioned at 45 °C prior to application, a general reduction in insecticidal efficacy was observed in wild-type and empty vector strains, as indicated by increased LC_50_ and LC_90_ values, with empty vector showing the greatest decline in potency. In contrast, the *PdDHN1*-transformed strain maintained relatively stable toxicity under thermal preconditioning (LC_50_ - 4.26 ± 0.20 and LC_90_ - 6.30 ± 0.29), indicating preservation of bioinsecticidal function despite elevated-temperature exposure of the bacterial formulation (**Table 2**). Across both preconditioning regimes, the toxicity remained consistent, demonstrating that *PdDHN1* expression enhances the thermal tolerance of *B. thuringiensis* insecticidal activity under standardized insect assay conditions.

Adult susceptibility to *B. thuringiensis* was evaluated following exposure to formulations preconditioned at either 28 °C or 45 °C, with all bioassays conducted under standardized conditions at 25 °C. Probit analysis confirmed significant dose–response relationships across all strains and treatments (p< 0.001), indicating consistent toxicity patterns. At 28 °C preconditioning, strain-dependent differences in insecticidal potency were observed (**Table 3**). The highest toxicity was observed in the *PdDHN1*-transformed strain, having the lowest LC_50_ (4.14 ± 0.19) and LC_90_ (5.98 ± 0.27). An intermediate susceptibility was found for the wild-type strain with LC_50_ (5.34 ± 0.22) and LC_90_ (8.02 ± 0.43), while the empty vector strain had LC_50_ of 6.05 ± 0.38 and LC_90_ of 9.35 ± 0.64, requiring higher concentrations for equivalent mortality.

**Table 3:** Toxicity of *B. thuringiensis* strains on adult RPW under normal and heat stress conditions.

Strain	Temp	Stage	LC_50_	LC_90_	LC_50_ CFU	LC_90_ CFU
WT	28	Adult	5.34 ± 0.22	8.02 ± 0.43	2.19×10^5^	1.05×10^8^
WT	45	Adult	6.29 ± 0.27	9.51 ± 0.66	1.94×10^6^	3.20×10^9^
ET	28	Adult	6.05 ± 0.38	9.35 ± 0.64	1.12×10^6^	2.24×10^9^
ET	45	Adult	7.25 ± 0.40	10.58 ± 0.99	1.78×10^7^	3.80×10^10^
DHN	28	Adult	4.14 ± 0.19	5.98 ± 0.27	1.38×10^4^	9.64×10^5^
DHN	45	Adult	4.45 ± 0.19	6.46 ± 0.29	2.81×10^4^	2.86×10^6^

When *B. thuringiensis* formulations were preconditioned at 45 °C prior to application, strain-specific divergence in toxicity profiles became more evident in adult insects. The wild-type and empty vector strains exhibited reduced insecticidal potency, as indicated by increased LC_50_ and LC_90_ compared to the 28 °C, with empty vector showing the greatest decline in efficacy. The *PdDHN1*-transformed strain also showed slightly reduced toxicity at 45 °C, with higher LC_50_ (4.45 ± 0.19) and LC_90_ (6.46 ± 0.29) values than at 28 °C but remained substantially more toxic than the wild-type and empty vector strains. These results indicate that *PdDHN1* expression preserved relatively high bioinsecticidal activity following thermal stress exposure of the formulation (**Table 3**). Within both preconditioning regimes, the *PdDHN1*-transformed strain maintained the highest toxicity, demonstrating relatively stable insecticidal performance and thermal tolerance of *B. thuringiensis* efficacy against *R. ferrugineus*.

### 3.6 Survival patterns of *B. thuringiensis*-treated RPW pests

Kaplan–Meier survival assays were conducted using a standardized concentration of 6.5 × 10^6^ CFU/mL, selected as a representative dose based on LC_50_-derived toxicity ranges to ensure measurable survival differences among wild-type, empty vector, and *PdDHN1*-transformed *B. thuringiensis* formulations.

#### 3.6.1 Survival of *B. thuringiensis*-treated RPW at the larval stage

Larval survival of *R. ferrugineus* was assessed following exposure to *B. thuringiensis* formulations that had been preconditioned under different thermal regimes, while all insect bioassays were conducted under identical optimal conditions (25 °C). Kaplan–Meier survival analysis revealed significant differences among treatments depending on the prior thermal exposure of the bacterial strains. With the 28 °C preconditioning of *B. thuringiensis* cultures, larval survival declined progressively across all treatments (log-rank test, p = 0.0166). However, no significant decline was observed for the untreated and buffer applied insects. The *PdDHN1*-transformed *B. thuringiensis* formulation induced a faster decline in larval survival, reaching 35.5% by day 9 (**Figure 5A**). In comparison, larvae treated with empty vector and wild-type *B. thuringiensis* preparations retained higher survival at the same time point (65.5% and 54.8%, respectively), indicating a slower onset of toxic activity in these groups. Over time, survival curves diverged further, with the *PdDHN1*-transformed *B. thuringiensis* treatment achieving near-complete mortality by day 18, whereas wild-type and empty vector *B. thuringiensis* formulations declined more gradually and approached full mortality only by day 21.

**Figure 5:**
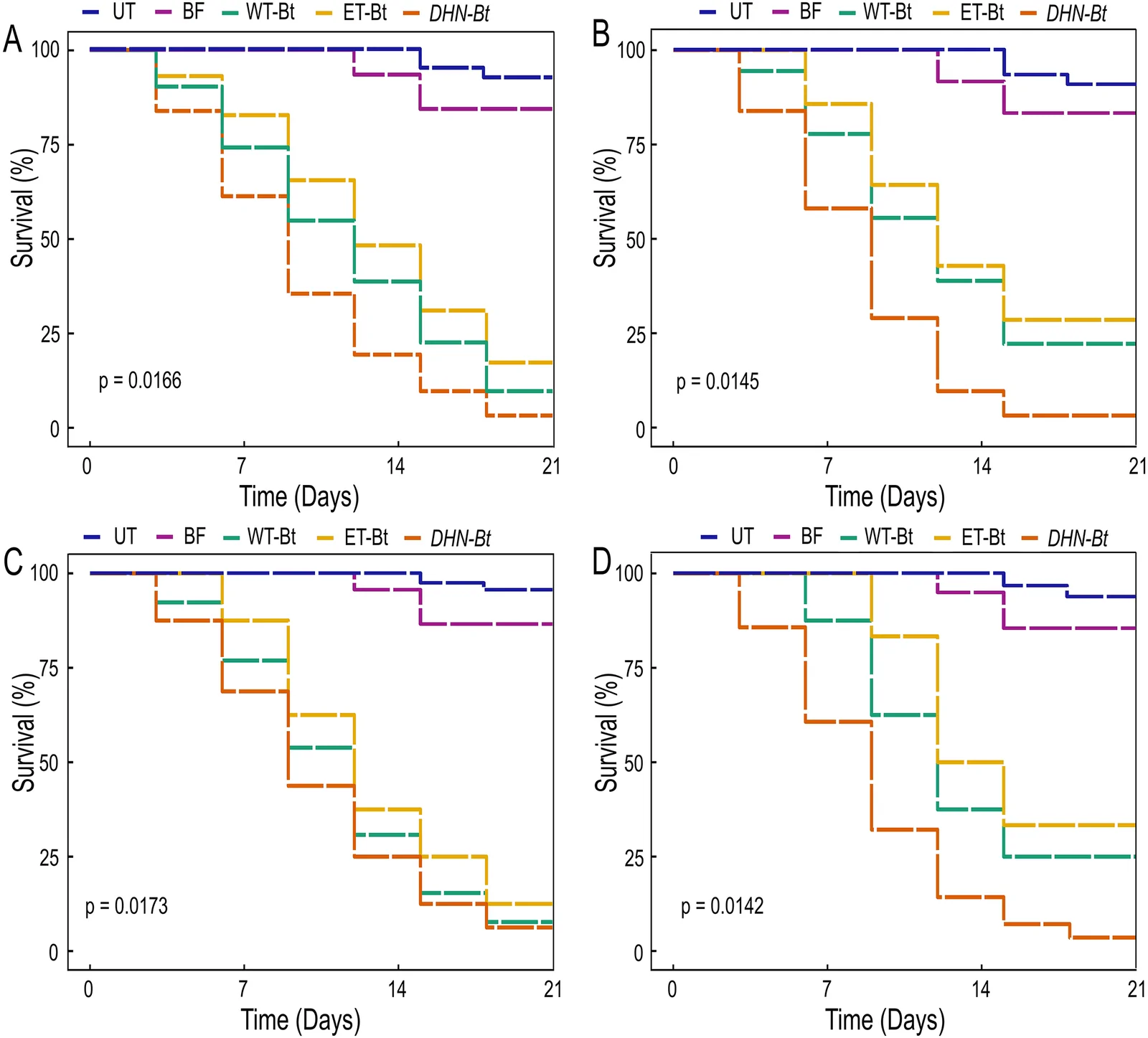
Kaplan–Meier survival analysis of Bt-treated RPW pests under different temperature conditions. Larval survival at **(A)** normal and **(B)** heat stress conditions. Adult survival at **(C)** normal temperature and **(D)** heat stress conditions. Survival curves represent time-to-mortality changes of wild-type WT-Bt, ET-Bt, and DHN-Bt treatments. Differences among groups were assessed using the log-rank test at p < 0.05. UT- untreated condition, BF- buffer treated condition, WT-Bt- wild-type Bt treated condition, ET-Bt- empty plasmid treated condition, and *DHN*-Bt- PdDHN1-transformed Bt condition.

A similar pattern was observed when *B. thuringiensis* cultures were preconditioned at 45 °C prior to application (log-rank test, p = 0.0145), but with a stronger separation among survival trajectories. The *PdDHN1*-trasnformed *B. thuringiensis* formulation maintained insecticidal performance despite thermal pre-exposure, reducing larval survival to 29.0% by day 9 and to below 10% by day 12. In contrast, wild-type *B. thuringiensis* and empty vector *B. thuringiensis* formulation showed similar delayed activity, maintaining higher survival at corresponding time points (approximately 55% to 64% at day 9) (**Figure 5B**). This shows that thermal preconditioning of *B. thuringiensis* strains significantly influences their subsequent insecticidal efficacy under standardized application conditions.

#### 3.6.2 Survival of *B. thuringiensis*-treated adult RPW

Kaplan–Meier survival analysis of adult *R. ferrugineus* revealed that prior thermal preconditioning of *B. thuringiensis* formulations had slight significant effect on overall survival when bacteria were conditioned under optimal conditions (28 °C) prior to application (log-rank test, p = 0.629). Under these conditions, wild-type, empty vector, and *DHN*-transformed *B. thuringiensis* treatments exhibited largely overlapping survival trajectories, with gradual and similar declines throughout the observation period (**Figure 5C**). Although the *PdDHN1*-expressing formulation showed a marginally earlier reduction in survival at initial time points, these differences were statistically distinct survival patterns, and all treatments converged toward similarly low survival levels by the end of the assay. The untreated and buffer applied insects showed no significant mortality during the 21-day period.

When Bt cultures were preconditioned at heat stress of 45 °C prior to application, adult survival trajectories diverged significantly (log-rank test, p = 0.0425). The *PdDHN1*-transformed *B. thuringiensis* formulation induced an accelerated mortality response, with survival decreasing to 60.7% by day 6, 32.1% by day 9, and 14.3% by day 12. In comparison, wild-type and empty vector *B. thuringiensis* maintained higher survival during the same period, with wild-type *B. thuringiensis* retaining 87.5% and 64.3% at days 6 and 9, respectively, while empty vector *B. thuringiensis* showed 83.3% and 50.0% survival at the corresponding time points (**Figure 5D**).

### 3.7 Temperature-dependent effects of *B. thuringiensis* strains on feeding efficiency parameters in larval and adult RPW

#### 3.7.1 Feed conversion efficiency in the larval stage of RPW

Larval FCE was evaluated following exposure to *B. thuringiensis* formulations preconditioned at either 28 °C or 45 °C, with all bioassays conducted under standardized conditions. Under the 28 °C preconditioning, significant differences in FCE were observed among *B. thuringiensis strains* and untreated and buffer applied RPW. The PdDHN*1*-transformed strain showed only a marginal reduction relative to wild-type *B. thuringiensis* (3.57%), indicating similar feeding efficiency under non-stress conditions (**Figure 6A**).

**Figure 6:**
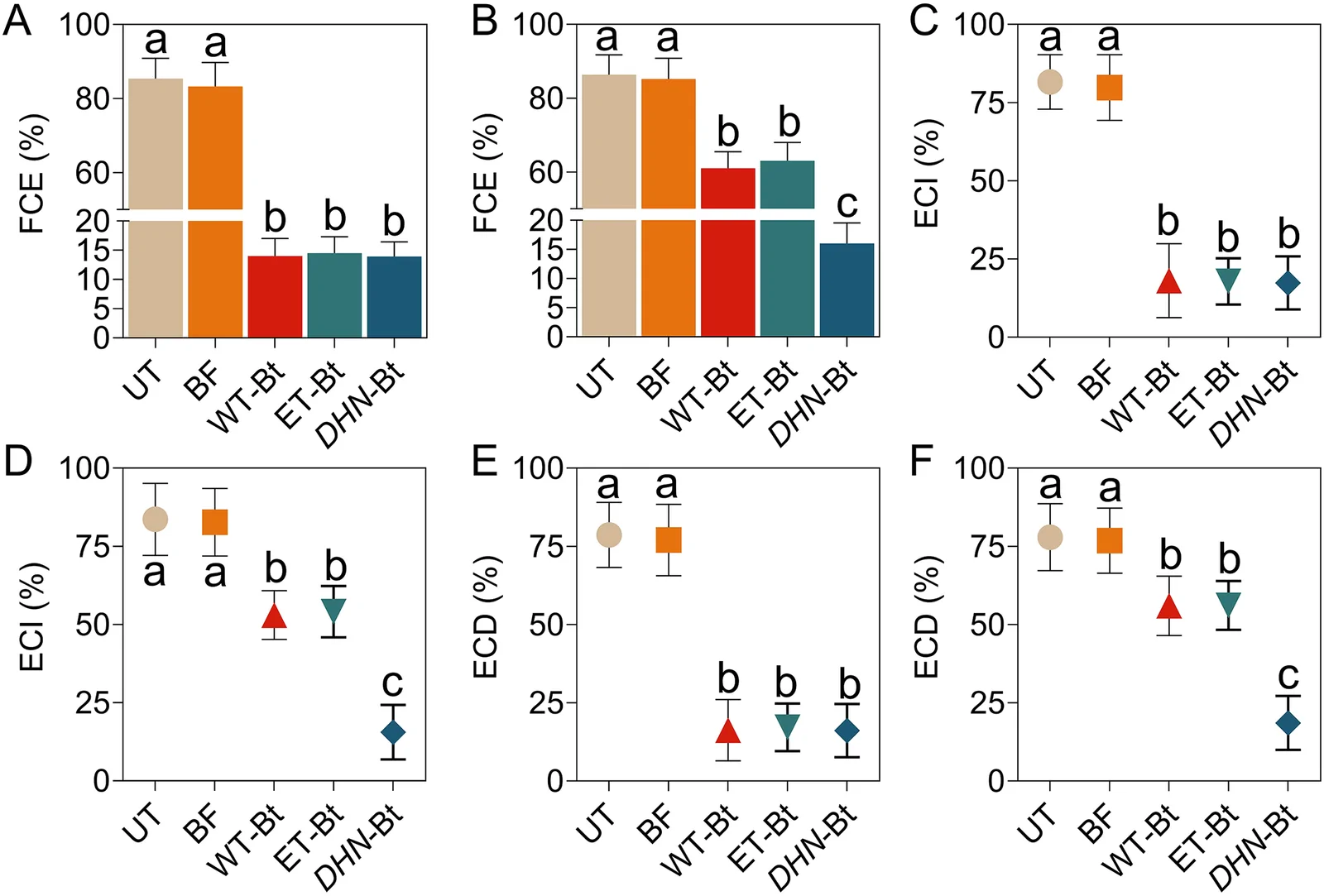
Effect of *B. thuringiensis* strains on the feeding efficiency of larval RPW. RPW larval FCE with **(A)** 28 °C and **(B)** 45 °C preconditioned *B. thuringiensis* formulation. RPW larval ECI with **(C)** 28 °C and **(D)** 45 °C preconditioned *B. thuringiensis* formulation. RPW larval ECD with **(E)** 28 °C and **(F)** 45 °C preconditioned *B. thuringiensis* formulation. Values are the mean ± SE. Different letters indicate statistically significant differences among treatments (p < 0.05), based on one-way ANOVA followed by Tukey’s *post hoc* test. UT- untreated condition, BF- buffer treated condition, WT-Bt- wild-type Bt treated condition, ET-Bt- empty plasmid treated condition, and *DHN*-Bt- PdDHN1-transformed Bt condition.

In contrast, treatment-dependent effects emerged following 45 °C preconditioning. While the wild-type and empty-vector strains maintained comparable FCE levels, the *PdDHN1*-expressing strain showed a significantly lower feeding efficiency, corresponding to 73.72% of the wild-type value (**Figure 6B**). This reduction was significantly greater than that observed in the empty vector treatment (p< 0.001), indicating a strong impairment of larval nutrient utilization associated specifically with the preconditioned *PdDHN1*-transformed *B. thuringiensis* formulation. These results indicate that *PdDHN1* expression was associated with a greater reduction in larval feeding efficiency following elevated-temperature preconditioning of *B. thuringiensis*.

#### 3.7.2 Efficiency of conversion of ingested food in the larval stage of RPW

Assessment of larval ECI revealed a specific dependence on the thermal preconditioning history of *B. thuringiensis* formulations. Under 28 °C preconditioning, ECI values remained tightly clustered across strains, with wild-type (18.05%), empty vector (17.83%), and *PdDHN1*-expressing strains (17.35%) showing statistically significant variation with the untreated and buffer applied insects (**Figure 6C**). This indicates that basal nutrient assimilation efficiency in larvae was largely unaffected when bacterial formulations were maintained under optimal growth conditions.

A different response was found following 45 °C preconditioning of *B. thuringiensis* cultures. In this context, wild-type and empty vector treatments maintained similar ECI (52.98% and 54.11%, respectively), suggesting a conserved pattern of nutrient conversion despite thermal exposure of the bacterial preparations (**Figure 6D**). In contrast, larvae exposed to the *PdDHN1*-transformed formulation exhibited a sharp decline in ECI (15.55%), representing reductions of 70.65% and 71.26% relative to wild-type and empty vector treatments, respectively. This suppression of conversion efficiency was statistically significant (p< 0.001), indicating a strain-specific disruption of nutrient assimilation under thermally preconditioned conditions.

#### 3.7.3 Efficiency of conversion of digested food in the larval stage of RPW

Patterns of ECD further showed the differential physiological impact of thermally preconditioned *B. thuringiensis* formulations on larval metabolism. When *B. thuringiensis* cultures were maintained at 28 °C prior to application, ECD remained statistically indistinguishable across treatments except for untreated and buffer applied RPW, indicating that digestive conversion processes were largely preserved under non-stress conditions (**Figure 6E**). Only minor deviations were observed, with the *PdDHN1*-transformed strain showing negligible reductions relative to wild-type and empty vector formulations.

Following 45 °C preconditioning, a divergence was found in the digestive efficiency. Larvae exposed to wild-type and empty vector treatments exhibited nearly identical ECD values (55.98% and 56.12%, respectively), suggesting limited impact of thermal preconditioning on their functional performance. In contrast, the *PdDHN1*-transformed formulation resulted in a decline in ECD (18.55%), corresponding to reductions of 66.86% and 66.95% relative to wild-type and empty vector treatments, respectively (**Figure 6F**). This effect was highly significant (p< 0.001) and indicates a substantial disruption of post-digestive nutrient conversion processes specifically associated with the *PdDHN1*-transformed strain under thermally preconditioned conditions. The ECD response follows the pattern observed for FCE and ECI, with the *PdDHN1*-transformed treatment showing lower nutrient conversion efficiency following elevated-temperature preconditioning.

#### 3.7.4 Feed conversion efficiency in adult RPW

Adult feeding performance remained largely unaltered when exposed to *B. thuringiensis* formulations preconditioned at 28 °C, with FCE values showing minimal dispersion across treatments (WT: 16.23%, EV: 17.17%, and DHN: 16.11%). The slight elevation observed in the empty vector treatment relative to wild-type (5.79%) did not show a meaningful change, indicating that adult nutrient assimilation efficiency is stable under non-stress formulation conditions (**Figure 7A**). However, the *B. thuringiensis* strain treatment differed significantly (p< 0.01) compared to untreated and buffer applied insects.

**Figure 7:**
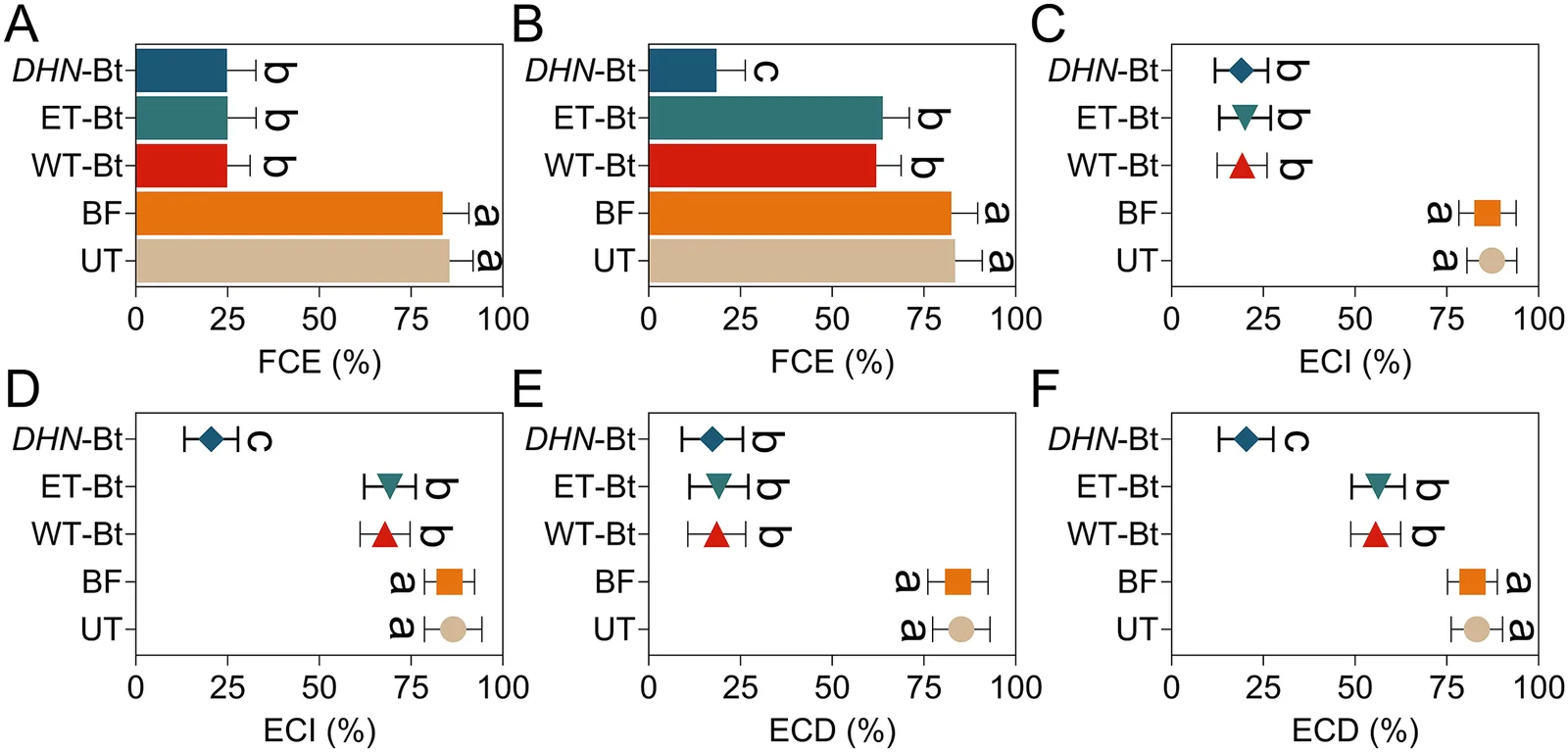
Effect of *B. thuringiensis* strains on the feeding efficiency of adult RPW. FCE with **(A)** 28 °C and **(B)** 45 °C preconditioned *B. thuringiensis* formulation. ECI with **(C)** 28 °C and **(D)** 45 °C preconditioned *B. thuringiensis* formulation. ECD with **(E)** 28 °C and **(F)** 45 °C preconditioned *B. thuringiensis* formulation. Values are the mean ± SE. Different letters indicate statistically significant differences among treatments (p < 0.05), based on one-way ANOVA followed by Tukey’s *post hoc* test. UT- untreated condition, BF- buffer treated condition, WT-Bt- wild-type Bt treated condition, ET-Bt- empty plasmid treated condition, and *DHN*-Bt- PdDHN1-transformed Bt condition.

A shift in response emerged following 45 °C preconditioning of *B. thuringiensis* cultures, revealing a strong treatment-dependent effect on adult feeding efficiency. While wild-type and empty vector formulations retained comparable FCE values (55.98% and 56.12%, respectively), adults exposed to the *PdDHN1*-transformed strain exhibited a reduction in FCE (18.55%) (**Figure 7B**). This represents a decline of approximately 66.86% relative to wild-type and 66.95% relative to the empty vector treatment, with differences being highly significant (p< 0.001). This substantial reduction indicates that the *PdDHN1*-transformed formulation exerts an inhibitory effect on nutrient conversion efficiency in adults when *B. thuringiensis* is subjected to elevated-temperature preconditioning. The consistency of this response with the larval-stage pattern indicates that the *PdDHN1*-transformed treatment was associated with reduced feeding efficiency in both developmental stages following elevated-temperature preconditioning.

#### 3.7.5 Efficiency of conversion of ingested food in the RPW adult stage

Adult ECI responses remained constrained when *B. thuringiensis* formulations were preconditioned at 28 °C, with values for wild-type (19.25%), empty vector (20.05%), and *PdDHN1*-expressing strains (19.95%) showing close agreement and statistically significant differences with untreated and buffer treatments (**Figure 7C**). This indicates that ingestion-to-biomass conversion efficiency in adults is largely unaffected under optimal formulation conditions.

Following 45 °C preconditioning, treatment-specific effects were observed. Wild-type and empty vector formulations exhibited comparable ECI values (52.98% and 54.11%, respectively), with only a marginal, non-significant increase in the empty vector group. In contrast, adults exposed to the *PdDHN1*-transformed formulation showed a substantial reduction in ECI (15.55%), corresponding to decreases of 70.65% relative to wild-type and 71.26% relative to the empty vector treatment (**Figure 7D**). This decline was highly significant (p< 0.001), indicating a disruption of ingested food conversion efficiency associated specifically with the *PdDHN1*-transformed strain under heat stress preconditioning. These results indicate that, while baseline ingestion efficiency remained relatively similar across bacterial treatments, the *PdDHN1*-transformed treatment was associated with a greater reduction in nutrient conversion efficiency following elevated-temperature preconditioning, consistent with the pattern observed in larvae.

#### 3.7.6 Efficiency of conversion of digested food in the RPW adult stage

Digestive conversion efficiency in adults exhibited strong stability under non-stress formulation conditions, with ECD remaining closely aligned among wild-type (18.56%), empty vector (19.17%), and *PdDHN1*-expressing strains (17.37%) following 28 °C preconditioning of *B. thuringiensis* (**Figure 7E**). The absence of statistically significant differences indicates that post-digestive nutrient processing is largely conserved across treatments when formulations are maintained under optimal conditions. However, they differed significantly (p< 0.001) with the untreated and buffer treated RPW insects.

Thermal preconditioning at 45 °C, resulted in a divergence in adult ECD responses. Wild-type and empty vector treatments retained similar efficiency (55.63% and 56.30%, respectively), suggesting limited functional disruption despite prior heat exposure of the bacterial formulations. In contrast, the *PdDHN1*-transformed strain induced a substantial reduction in ECD (20.36%), corresponding to decreases of 63.40% relative to wild-type and 63.84% relative to the empty vector control (**Figure 7F**). This effect was highly significant (p< 0.001) and reflects an impairment of post-digestive nutrient conversion processes in adults exposed to the *DHN*-modified formulation. The *PdDHN1*-transformed treatment was associated with reduced post-digestive nutrient conversion efficiency following thermal preconditioning, consistent with the response observed in larvae.

### 3.8 Survival and nutritional indices association with thermally preconditioned *B. thuringiensis* strains

Correlation analysis revealed distinct patterns among survival, FCE, ECI, and ECD across different temperature preconditioning regimes and life stages. At 28 °C preconditioning applied to larvae, survival showed a significant positive correlation with FCE and ECI, indicating that higher food conversion efficiencies are associated with improved survival under moderate thermal conditions (**Figure 8A**). ECD also followed a similar positive trend, although with comparatively weaker significance. These relationships indicate an association between survival and nutritional efficiency under the 28 °C preconditioning condition. Similarly, under 45 °C preconditioning applied to larvae, the relationships were significant (p< 0.001) (**Figure 8B**). Survival exhibited positive correlations with FCE, ECI, and ECD, reflecting a consistent low–low and high–high trend even under thermal stress conditions.

**Figure 8:**
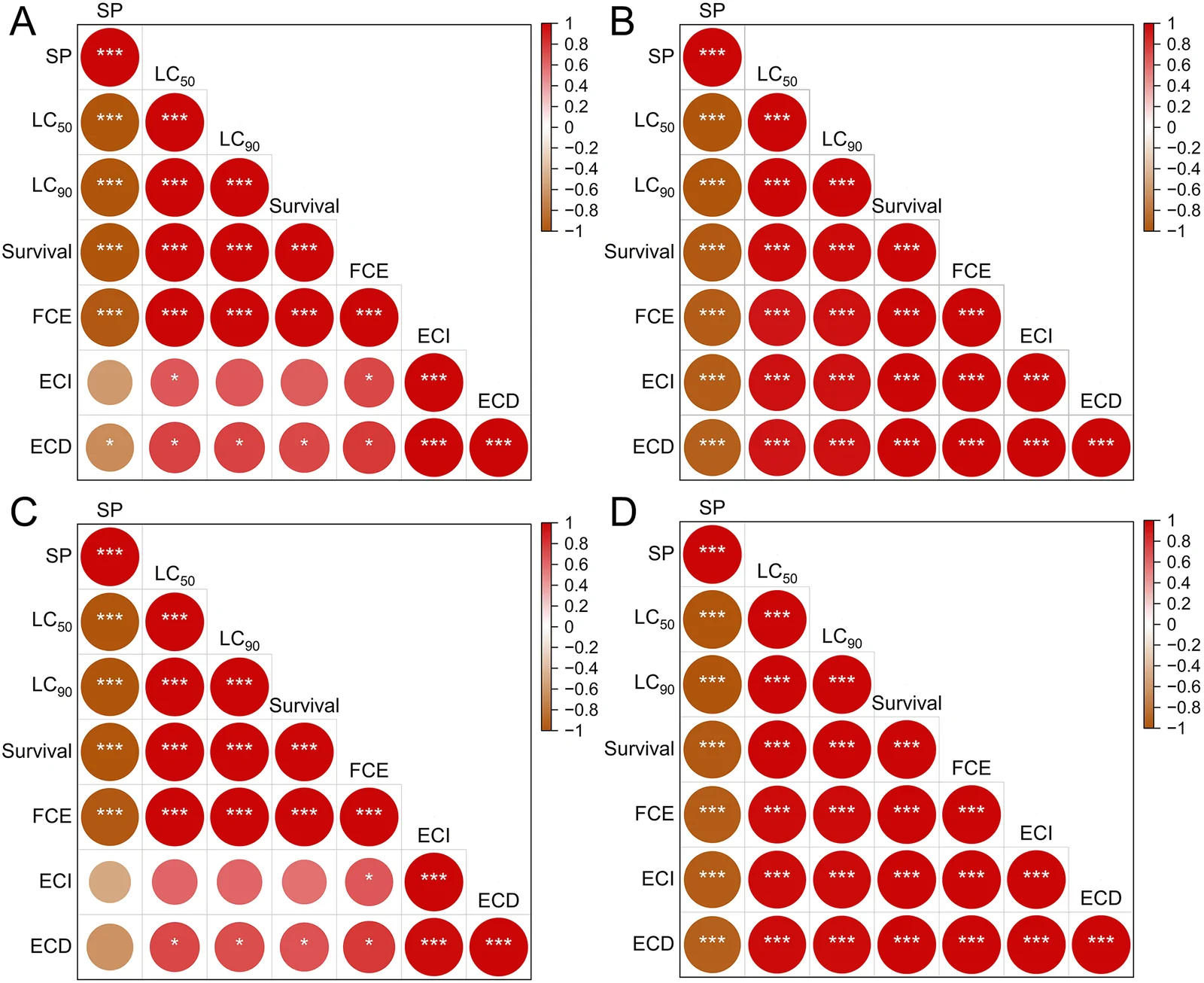
Correlation among survival, FCE, ECI, and ECD under different thermal preconditioning treatments. *B. thuringiensis* preconditioned at **(A)** 28 °C applied to larva, **(B)** 45 °C applied to larvae, **(C)** 28 °C applied to adults, and **(D)** 45 °C applied to adults. SP- spore production, FCE- feeding conversion efficiency, ECI- efficiency of conversion of ingested food, and ECD- efficiency of conversion of digested food. Statistical significance is shown by * at p< 0.05,** at p< 0.01, and *** at p< 0.001.

For adult-stage preconditioning at 28 °C, correlations resembled those observed in larval stages at the same temperature. Survival maintained a positive association with FCE and ECI under the 28 °C preconditioning condition (**Figure 8C**). ECD further showed a positive but less effective relationship. In adults preconditioned at 45 °C, survival also exhibited positive correlations with FCE, ECI, and ECD (**Figure 8D**). This indicates that the positive associations between survival and nutritional efficiency were maintained under the 45 °C preconditioning condition.

## 4 Discussion

DHNs are well-characterized plant stress-associated proteins, but their application in microbial biocontrol systems remains poorly studied. Our findings show that heterologous expression of the date palm *PdDHN1* in *B. thuringiensis* can enhance its performance under thermal stress while maintaining insecticidal activity following thermal preconditioning. This provides a basis for using plant-derived stress-protective proteins to improve thermal tolerance and potential field stability of microbial biopesticides.

Microscopic examination of *B. thuringiensis* strains demonstrated that elevated temperature affected cellular structure, consistent with the effects of thermal stress on membrane organization, protein folding, and aggregation of intracellular components (Katikaridis et al., 2021). In agreement with this, previous studies have reported that thermal exposure can significantly reduce cellular integrity and diminish the persistence and stability of functional proteins in *B. thuringiensis* var. *kurstaki*, thereby limiting its bioinsecticidal efficiency under adverse environmental conditions (Hung et al., 2016). The *PdDHN1*-transformed *B. thuringiensis* strain exhibited improved cellular preservation under thermal conditions. This improved morphological stability is consistent with the functional properties of DHNs, which are widely recognized as molecular chaperones capable of stabilizing cellular membranes and proteins under abiotic stress (Abdul Aziz and Masmoudi, 2023). DHNs achieve this protective role by preventing protein aggregation, maintaining hydration shells around macromolecules, and preserving membrane integrity during stress exposure (Qin et al., 2026). Such protective mechanisms likely contribute to the maintenance of cellular organization observed in the recombinant strain, thereby supporting continued physiological functionality under conditions that are otherwise detrimental to bacterial survival (Khader et al., 2025; Abdul Aziz et al., 2025). Further supporting this interpretation, previous heterologous expression studies have shown that overexpression of the DHN-like gene *CsLEA11* from *Cucumis sativus* in *E. coli* significantly enhances cell viability and confers increased tolerance to heat stress, reinforcing the broader applicability of LEA/DHN proteins in microbial stress protection (Zhou et al., 2017).

The effects of prolonged thermal exposure on *B. thuringiensis* were evident at the level of sporulation, with elevated temperature resulting in reduced sporulation efficiency in the wild-type and empty-vector strains. These results were obtained using a controlled *in vitro* thermal preconditioning approach in which vegetative bacterial cells were exposed to the respective temperature conditions for 7 days. Therefore, the findings should be interpreted as an assessment of the intrinsic capacity of the strains to maintain growth and sporulation under prolonged heat stress rather than as a direct simulation of the natural *B. thuringiensis* life cycle or events occurring within the target insect. Previous studies have reported that elevated temperatures can adversely affect sporulation and toxin production in *B. thuringiensis* (Peng et al., 2019; Verma et al., 2024). Similarly, reduced viable spore counts under elevated production temperatures and inhibition of spore formation at temperatures approaching 40 °C have been documented (Zhou et al., 2008; Ignatenko et al., 1983). These findings support the use of controlled thermal exposure as an experimental approach for assessing the temperature sensitivity of *B. thuringiensis* strains.

In contrast, the *PdDHN1*-expressing strain maintained comparatively stable sporulation following heat treatment, indicating greater tolerance to the imposed thermal stress. This observation suggests that PdDHN1 expression may help maintain cellular processes associated with sporulation during prolonged exposure to elevated temperature. Given the reported ability of DHNs to protect cellular proteins against stress-induced aggregation, PdDHN1 may contribute to cellular protection under heat stress. Consistent with this possibility, DHN-5 containing conserved K-segments has been reported to enhance stress tolerance in *E. coli* through protection against protein aggregation (Drira et al., 2015). Thus, the improved sporulation observed in the PdDHN1-expressing strain following thermal preconditioning was accompanied by maintained biological performance in subsequent insect bioassays.

The total soluble protein analysis showed that the *PdDHN1*-expressing strain maintained a higher total soluble protein concentration following prolonged thermal exposure compared with the wild-type and empty-vector strains. This response may be associated with the protein-protective properties of DHNs, which have been reported to interact with and protect proteins and other cellular macromolecules under stress conditions (Smith and Graether, 2022). The maintenance of measurable total soluble protein content in the *PdDHN1*-expressing strain was associated with the preservation of its biological performance under heat stress.

RPW is a major pest of date palm species in arid and semi-arid regions, where persistently high temperatures may influence host–pathogen interactions (Masmoudi et al., 2021). The survival responses of RPW exposed to thermally preconditioned *B. thuringiensis* preparations showed strain- and stage-dependent, progressive mortality patterns consistent with established *B. thuringiensis*–insect interactions, in which Cry toxins are ingested, solubilized, and activated in the midgut, followed by epithelial disruption and physiological dysfunction. The reduced mortality observed for the wild-type and empty-vector strains following thermal preconditioning indicates that prolonged heat exposure affected the subsequent insecticidal performance of these preparations. This reduction may be associated with heat-induced changes in components contributing to Cry-mediated toxicity. In contrast, the sustained insecticidal activity of the *PdDHN1*-transformed strain suggests that *PdDHN1* expression may contribute to maintaining the biological performance of the bacterial preparation following prolonged heat exposure. The greater susceptibility of RPW larvae than adults may reflect differences in feeding intensity, gut physiology, and midgut susceptibility to Cry toxin action. Similar time- and stage-dependent mortality patterns have been reported in *Dioryctria abietella*, where *B. thuringiensis* exposure caused progressive mortality accompanied by changes in detoxification and antioxidant enzyme activities (Wang et al., 2025), and in *Ectomyelois ceratoniae*, where *B. thuringiensis* var. *kurstaki* caused gradual mortality that increased with exposure duration (Ismahane et al., 2025). The delayed mortality observed in the wild-type and empty-vector treatments following thermal preconditioning may reflect heat-induced changes in components contributing to their insecticidal activity. The enhanced performance of the *PdDHN1*-transformed strain may be associated with the intrinsically disordered nature of DHNs and their reported ability to interact with membranes, water molecules, and metal ions (Sabeem et al., 2022). Similar anti-aggregation and protein-protective roles have been reported for LEA proteins under dehydration and thermal stress conditions (Goyal et al., 2005). These properties provide a plausible basis for the improved biological performance of the *PdDHN1*-expressing strain following thermal preconditioning and support the potential of DHNs as biotechnological tools for enhancing the thermal tolerance and biological performance of *B. thuringiensis* under high-temperature conditions.

The observed alterations in host physiological performance further support the strain-dependent differences in the *B. thuringiensis* formulations. Under thermal preconditioning, the relatively high feed-utilization indices in wild-type and empty-vector treatments suggest limited disruption of digestive efficiency, whereas their suppression in the *PdDHN1*-expressing treatment indicates greater impairment of host metabolic processing. These changes in feeding and nutrient-conversion indices were consistent with the physiological effects commonly associated with *B. thuringiensis* exposure, including disruption of midgut function and nutrient utilization in susceptible insects. *B. thuringiensis*-induced impairment of digestive physiology is associated with epithelial damage, disruption of brush-border membrane integrity, and altered enzyme activity, ultimately affecting food utilization and growth (Shamim et al., 2024). Consistent with this, sublethal *B. thuringiensis* exposure reduced nutritional indices and altered immune-related enzyme activities in *Tuta absoluta* (Khatami et al., 2023), while *B. thuringiensis* var. *kurstaki*, alone or combined with botanical insecticides, enhanced toxicity, suppressed detoxification enzymes, and reduced pupal weight in *Hyphantria cunea* (Oftadeh et al., 2026).

Additional evidence from coleopteran systems, including the common European cockchafer and white grubs (Coleoptera: Scarabaeidae), supports the effectiveness of *Bacillus*-based agents, with mortality mediated through midgut receptor binding and proteolytic activation of Cry toxins (Selvakumar et al., 2007; Sushil et al., 2008; Sezen et al., 2007). The Cry protoxin is activated into an approximately 60-kDa toxin by midgut proteases and subsequently binds receptors on brush-border membrane vesicles, causing pore formation, epithelial swelling, and eventual lysis of gut tissue (Muita and Baxter, 2023). This disruption can lead to septicemia and starvation, contributing to insect mortality. The sustained insecticidal activity observed with *PdDHN1* expression following thermal preconditioning indicates that the biological performance of the bacterial preparation was maintained despite heat exposure. More broadly, the strain-dependent responses observed indicate that *B. thuringiensis* biological performance under elevated temperature involves multiple cellular and physiological responses to thermal stress. Thus, incorporating stress-protective elements such as *PdDHN1* represents a potential molecular strategy for improving the stability and consistency of *B. thuringiensis*-based biocontrol agents under elevated-temperature conditions.

## 5 Conclusion

Heterologous expression of the date palm DHN gene *PdDHN1* in *B. thuringiensis* serovar *tenebrionis* enhanced the functional tolerance of the bacterium under thermal preconditioning, resulting in improved maintenance of sporulation and sustained insecticidal performance relative to wild-type and empty vector strains. Across bioassays, the *PdDHN1*-transformed formulation consistently exhibited enhanced biological efficacy, including lower lethal concentration and accelerated mortality of *R. ferrugineus* under standardized post-application conditions. In addition, *PdDHN1* expression induced coordinated physiological disruptions in the host insect, affecting ingestion, assimilation, and post-digestive nutrient conversion processes. This was evidenced by significant reductions in FCE, efficiency of ingested food utilization, and efficiency of digested food conversion, particularly following thermally preconditioned bacterial treatments. These responses indicate changes in feeding and nutrient utilization following exposure to the bacterial treatments. The observed effects may be associated with the stress-protective properties of DHNs and their potential contribution to maintaining protein and cellular functions under elevated temperature. The feeding assay provided a controlled means of evaluating the oral toxicity of the B. thuringiensis strains, its application to field conditions requires further consideration because RPW larvae remain concealed within the palm tissues and are consequently less accessible to externally applied insecticides. Future studies should therefore investigate delivery strategies capable of reaching larvae within infested palms, while adult-targeted approaches, including attractive bait-based formulations, may provide an additional route for field management. Although further molecular validation is required, the observed responses indicate that *PdDHN1* expression was associated with maintenance of bacterial biological performance and insecticidal activity following thermal preconditioning. Future work should investigate the molecular basis of the observed thermal tolerance, including direct assessment of Cry3A activity and protein stability under controlled temperature conditions, alongside midgut-targeted mechanistic studies, field evaluation, and formulation development to assess its applicability in sustainable pest management systems. Expressing a date-palm *DHN* gene could hypothetically improve vegetative-cell survival under desiccation or other environmental stresses. However, enhanced persistence of the transformed strain has not been demonstrated and cannot be inferred from *DHN* function alone. Prolonged exposure could affect palm- and soil-associated microbial communities and susceptible non-target organisms, but evaluating these risks requires dedicated microbiome and ecotoxicological studies. This study provides an approach for improving the performance of *B. thuringiensis*-based biopesticides in high-temperature environments, where thermal instability typically limits efficacy in sustainable pest management.

## Data Availability

The original contributions presented in the study are included in the article/supplementary material. Further inquiries can be directed to the corresponding author.
